# A high-performance, portable, field-deployable gas analyzer

**DOI:** 10.1016/j.ohx.2026.e00792

**Published:** 2026-05-20

**Authors:** Mark B. McKinnon, Ian Brady

**Affiliations:** UL Research Institutes, Fire Safety Research Institute, Columbia, MD, United States

**Keywords:** Gas analyzer, Combustion, Chemistry, Field testing, Fire testing

## Abstract

Large-scale fire experiments sometimes require measurement of gas concentrations at many locations to provide insight about fire dynamics and the influence of combustion and oxygen availability on the evolution of structure fires. These experiments often take place in the field which may limit accessibility and introduce additional logistical complications related to collecting data on the gas composition at sampling locations throughout the structure. The sensor module that is the subject of this work was designed to simplify high fidelity field measurement of gas concentrations. The major process improvements in the presented design include portability, reduction of necessary infrastructure, and removal of reliance on proprietary hardware and software. This gas sensor module incorporates a paramagnetic oxygen sensor, a non-dispersive infrared carbon dioxide sensor, and a non-dispersive infrared carbon monoxide detector into a single, small form-factor carrying case and relies on a single cable connection for both power and communications. All design files and processing scripts are presented and the sensor module is characterized in this manuscript.

## Graphical abstract

**Figure:**
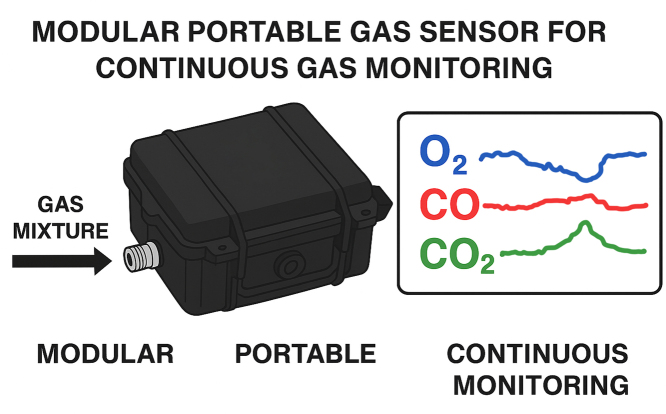

## Specifications table

**Table:** 

Hardware name	Portable gas sensor module
Subject area	Engineering and material science
Hardware type	Field measurements and sensors

Closest commercial analog	The closest commercial analogs are rack-mount gas analyzers and portable multi-gas meters.

Open source license	CERN Open Hardware Version 2.0 - Permissive
Cost of hardware	$4924.88
Source file repository	https://zenodo.org/doi/10.5281/zenodo.18168325

## 1 Hardware in context

This sensor module incorporates a paramagnetic oxygen sensor, a non-dispersive infrared carbon dioxide sensor, and a non-dispersive infrared carbon monoxide detector into a single, small form-factor carrying case. These sensing technologies allow for high accuracy and stability over a wide measurement range. Commercially-available alternatives that incorporate these sensing technologies and the corresponding measurement ranges are typically rack-mounted, which limits the portability of the sensors and necessitates plumbing sampled gases from the measurement point to the analyzer rack. Alternative portable sensors may be handheld, but most often rely on less expensive, smaller form factor sensing technologies that are less accurate and stable, are responsive to smaller gas concentration ranges, and more sensitive to interference from a larger variety of gases.

## 2 Hardware description

**Fig. 1:**
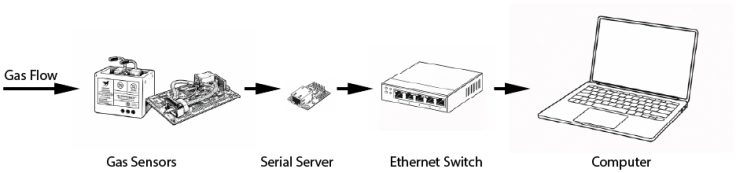
Schematic representation of gas sensor module system.

The sensor module presented here was designed to simplify high fidelity field measurement of gas concentrations primarily in combustion and fire science-related experiments. A schematic representation of the major components that shows the flow of data within the designed system is provided as Fig. 1. The schematic shows the philosophy behind the module design. This involves an uncharacterized gas flowing to the gas sensors, communication between the gas sensors and serial servers, a reduction in the number of cables passing information via the ethernet switch, and a single ethernet cable passing all necessary data from the gas sensors to a computer.

**Fig. 2:**
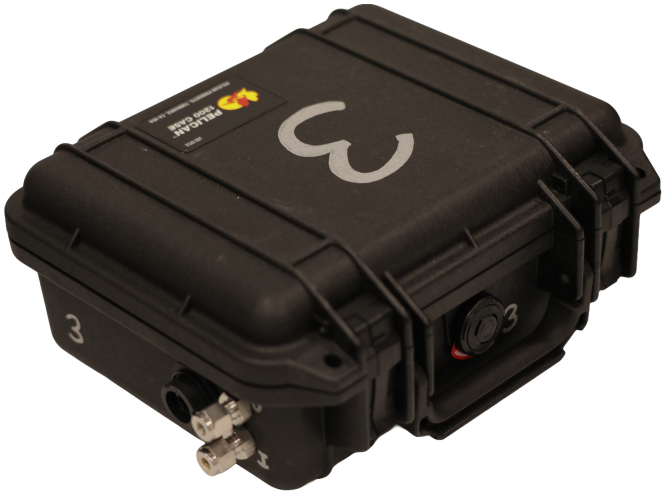
Sensor module with lid closed.

**Fig. 3:**
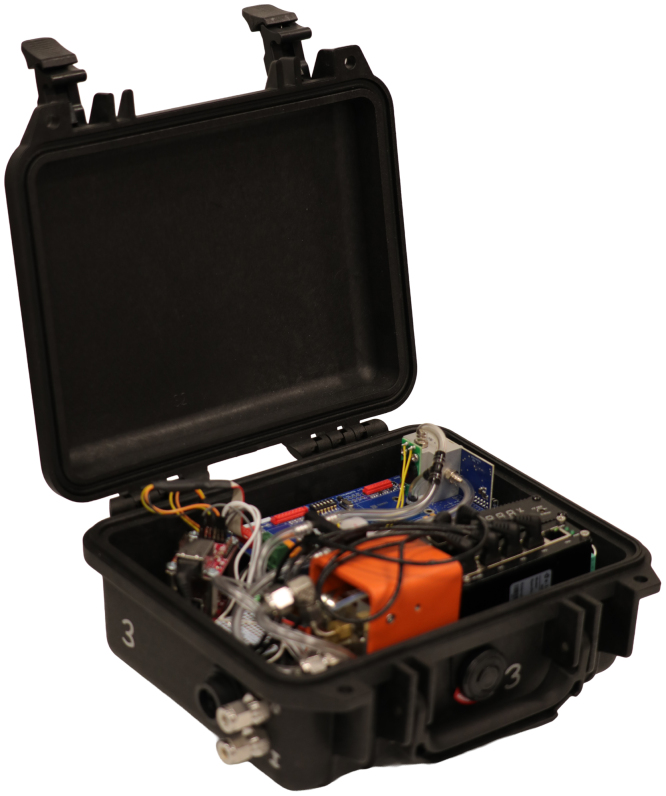
Sensor module with lid open.

Photographs of the sensor module are included in Fig. 2, Fig. 3. Large-scale fire experiments sometimes require measurement of gas concentrations at many locations to provide insight about fire dynamics and the influence of combustion and oxygen availability on the evolution of a structure fire [1], [2]. These experiments often take place in the field which may limit accessibility and introduce additional logistical complications related to collecting data on gas composition. The major process improvements this design targeted included portability, reduction of necessary plumbing, reduction of cables for data and power transmission, and removal of reliance on proprietary hardware and software.

**Fig. 4:**
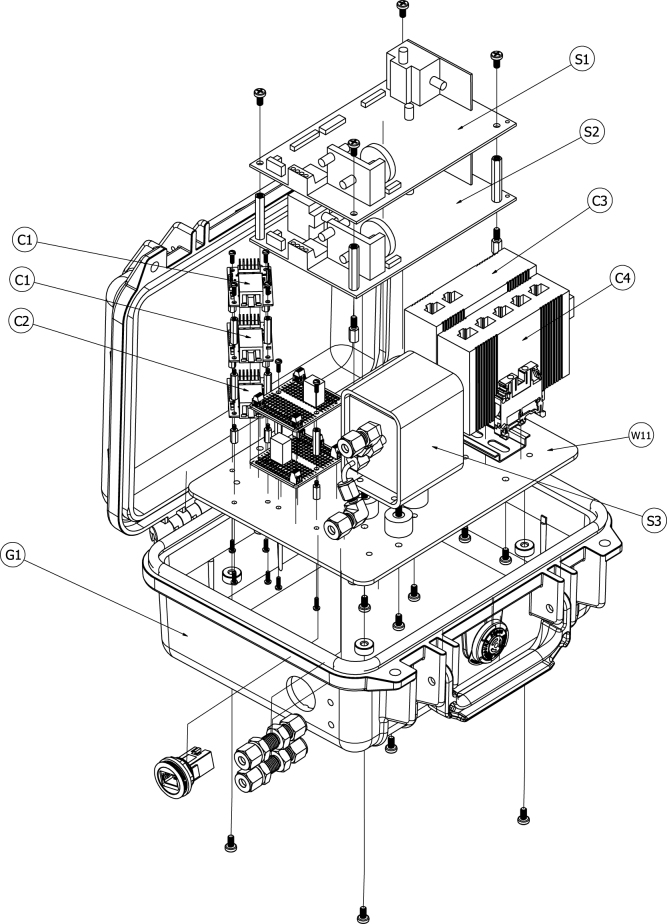
Exploded view of sensor module.

Fig. 4 shows an exploded view of all components of the sensor module. OEM gas sensors with small form factors were specified in the design of this module to reduce the overall footprint of the sensor module and to allow it to fit into an easily portable case. A paramagnetic oxygen sensor is known to provide a more accurate and stable signal than electrochemical sensors, but paramagnetic sensors are often designed into analyzers that have large form factors, are rack mounted, and cannot be easily transported in the field [3]. A Hummingbird Premus Delta paramagnetic oxygen sensor with digital output was specified in this application. Non-dispersive infrared (NDIR) technology is a high fidelity method for quantification of carbon dioxide and carbon monoxide that is more accurate and stable than other measurement technologies. Edinburgh Instruments Gascard NG NDIR sensors with calibrated ranges from 0 to 10 vol.% for carbon dioxide and carbon monoxide were incorporated into the module.

The module was designed to include a means for communication and power transmission in a single cable with power-over-ethernet (PoE) technology. PoE technology reduces the cable infrastructure required to transmit sensor signals over long distances to a connected micro-controller or computer for data logging, visualization, and/or process control. This is a major improvement over existing infrastructure which would require separate power and data transmission cables. To facilitate the use of PoE technology, a PoE splitter, a 4-port ethernet switch, and three Wiznet serial servers were designed into the module. The sensors and the serial servers allow for digital input and output, so a separate data acquisition system suited for analog signals or reliant on proprietary software or hardware is not necessary and all calibrations may be completed via a computer interface.

The PoE splitter specified for the module is an industrial model that splits a PoE signal into data via an RJ45 (ethernet) jack and DC power via a terminal block. The PoE splitter RJ45 port is connected to the upload port of the ethernet switch via a category 6 A ethernet cable. Three of the ports on the ethernet switch are connected to the ethernet ports on the serial servers. The oxygen sensor is connected to a serial server designed to operate with the RS-232 communication protocol. The carbon dioxide and carbon monoxide sensors are connected to serial servers designed to communicate with the Transistor–Transistor Logic (TTL) serial communication protocol.

24VDC power transmission from the PoE splitter is connected to industrial terminal block leads on the ethernet switch and also passed to terminal blocks soldered to a breadboard. The breadboard includes circuits that split the 24VDC input into two outputs to power the NDIR sensors. The breadboard also includes DC–DC converters to reduce the voltage from 24VDC to 5VDC and 3.3VDC to power the paramagnetic sensor and the serial servers, respectively. The PoE splitter also provides a ground point on the terminal block. This ground point was connected to a grounded DIN rail-mounted terminal block. The DIN rail was mounted on an aluminum plate which served to ground all other powered equipment.

The smallest Pelican carrying case in which the sensors would fit was specified for the module to ensure portability of the module. Portability of the module facilitates deployment of the module closer to the measurement point, which eliminates the needs for a rack-mounted analyzer and typically reduces the plumbing infrastructure required to transport the sample gas to the analyzer. An aluminum plate was designed to fit in the Pelican case and a pattern of holes were milled out of the plate to allow for mounting of all components onto the plate prior to installation in the case. The plate had four tapped through holes that allowed machine screws to be inserted through the bottom of the case to secure the plate to the case.

Holes were drilled through the Pelican case to accommodate 1/4-inch bulkhead compression fittings for inlet and exhaust of sampled gases and a pass-through bulkhead fitting for the ethernet cable. The inlet compression fitting was connected to flexible tubing that terminated at a 1/4-inch to 1/8-inch compression reducing elbow. The 1/8-inch side of the reducing elbow was installed on the inlet tube of the paramagnetic sensor. An additional 1/4-inch to 1/8-inch reducing elbow was connected to the exhaust tube from the paramagnetic sensor. A 1/4-inch flexible tube connected the reducing elbow to a barbed fitting at the inlet to the carbon monoxide NDIR sensor. Flexible tubing was used to connect the exhaust from the carbon monoxide NDIR sensor to the inlet of the carbon dioxide NDIR sensor and from the exhaust of the carbon dioxide NDIR sensor to the exhaust 1/4-inch bulkhead fitting through the case. This consecutive flow plumbing design results in fast response times, small time offsets between sensor measurements, and ease of connection.

Hardware that is required for operation of the sensor module that have not been mentioned in the section include a PoE injector, ethernet cable, and a computer with an open ethernet port. A PoE injector accepts (or transmits) a data signal through an ethernet cable and passes power into the output ethernet cable. There are several PoE protocols and this work was done using the PoE++ protocol (IEEE 802.3bt Type 4) which is capable of passing up to 100 W over an ethernet cable. In operation, the sensor module typically draws a peak of approximately 20 W at start up and draws approximately 14 W during steady operation, so lower PoE power transmission protocols would likely be acceptable for this application. No other PoE protocols were tested with the sensor module, but PoE injectors and splitters that are compliant with IEEE 802.3at and IEEE 802.3bt Type 3 (up to 60 W) would likely provide the necessary power for full functionality of the sensor module.

The Collect_Gas_Data.py file is a Python script that is used to visualize and log the data measured by the sensor module. The script builds a dashboard in a web browser that updates at a rate of 2 Hz, plots real-time data in a chart, and visualizes the instantaneous measured gas concentrations in numeric indicators.

The script also generates a ‘raw_data_log.csv’ file with comma-separated data points collected from all the sensors in the module at a rate of 2 Hz. A major advantage of using Python for data visualization and data logging is that this approach does not limit the data acquisition to a specific operating system nor rely on proprietary data acquisition systems. Use of the ethernet protocol and Python data management scripts facilitate incorporation of many sensor modules into a network without significantly increasing the required infrastructure, which effectively simplifies increasing the number of sampling locations in a large-scale fire experiment or air quality assessment.

The highlights of this hardware are summarized as follows:

• Minimizes the footprint of instrumentation required for high fidelity measurement of oxygen, carbon monoxide, and carbon dioxide.
• Reduces plumbing, data, and power transmission infrastructure requirements for deployment of gas monitoring in field experiments.
• Provides data visualization and logging abilities in a open-source and operating system-agnostic way that facilitates scalability of the size of the sensor network.

## 3 Design files summary

The design files are summarized in Table 1. All design files and data acquisition scripts required for replication are available in a Zenodo repository [4] available under a CERN Open Hardware License (Version 2.0 - Permissive). All instructions for construction and setup of all systems are included in this manuscript.

**Table 1:** Design files.

Design filename	File type	Open source license	Location of the file
Sensor_Module_Assembly.stp	CAD file	CERN-OHL-P-2.0	https://zenodo.org/[4]
Mounting_Plate.stp	CAD file	CERN-OHL-P-2.0	https://zenodo.org/[4]
Plumbing_Diagram.pdf	Image	CERN-OHL-P-2.0	https://zenodo.org/[4]
Electrical_Diagram.pdf	Image	CERN-OHL-P-2.0	https://zenodo.org/[4]
Comms_Diagram.pdf	Image	CERN-OHL-P-2.0	https://zenodo.org/[4]

## 4 Bill of materials

The bill of materials for the sensor module is provided as Table 2. The ‘Source of materials’ column includes hyperlinks directly to individual product pages where possible.

**Table 2:** Bill of materials.

Designator	Component	Number	Cost per unit	Total cost	Source of materials	Material type
S1	NDIR CO2 Sensor	1	1134.00	1134.00	Edinburgh	Composite
S2	NDIR CO Sensor	1	1400.00	1400.00	Edinburgh	Composite
S3	O2 Sensor	1	1658.00	1658.00	Hummingbird	Metal
C1	RS-232 Serial Server	2	28.70	57.40	DigiKey	Composite
C2	TTL Serial Server	1	27.76	27.76	DigiKey	Composite
C3	PoE Splitter	1	99.00	99.00	amazon	Polymer
C4	Ethernet Switch	1	43.50	43.50	amazon	Polymer
C5	Ethernet Cable	5	8.59	42.95	amazon	Polymer
C6	Ethernet Bulkhead	1	10.64	10.64	DigiKey	Polymer
C7	Serial Comm Cable	2	3.39	6.78	DigiKey	Composite
C8	Heat Shrink	1	6.99	6.99	amazon	Polymer
P1	1/4${}^{\prime \prime }$ Bulkhead Fitting	2	30.69	61.38	McMaster-Carr	Metal
P2	1/4${}^{\prime \prime }$ Reducing Elbow	2	60.85	121.70	McMaster-Carr	Metal
P3	1/4${}^{\prime \prime }$ OD Tubing	1	12.00	12.00	McMaster-Carr	Polymer
W1	16 AWG Stranded Wire	2	5.83	11.66	McMaster-Carr	Composite
W2	DIN rail	1	4.13	4.13	McMaster-Carr	Metal
W3	Grounding terminal block	1	5.98	5.98	DigiKey	Composite
W4	Female-to-female jumper (12 in.)	1	3.95	3.95	DigiKey	Composite
W5	Female-to-female jumper (6 in.)	1	1.95	1.95	DigiKey	Composite
W6	1/4 size soldered breadboard	2	3.95	7.90	DigiKey	Composite
W7	Circuit Board Terminal Block	5	0.54	2.70	DigiKey	Composite
W8	Jumper Wire Kit	1	12.99	12.99	DigiKey	Composite
W9	DC–DC Step Down Regulator (3.3 V)	1	5.95	5.95	DigiKey	Composite
W10	DC–DC Step Down Regulator (5 V)	1	4.89	4.89	DigiKey	Composite
W11	Grounding Plate	1	24.96	24.96	Xometry	Metal
G1	Case	1	66.95	66.95	Pelican	Polymer
G2	F–M Standoff (M2.5 × 10 mm)	6	0.81	4.86	McMaster-Carr	Metal
G3	Standoff (M2.5 × 20 mm)	4	0.89	3.56	McMaster-Carr	Metal
G4	F–F Standoff (M2.5 × 20 mm)	6	0.74	4.44	McMaster-Carr	Metal
G5	F–M Standoff (M4 × 10 mm)	4	0.90	3.60	McMaster-Carr	Metal
G6	F–F Standoff (M4 × 35 mm)	4	2.20	8.80	McMaster-Carr	Metal
G7	Machine Screw (M2.5)	12	4.05	4.05	McMaster-Carr	Metal
G8	Machine Screw (M4)	15	5.80	5.80	McMaster-Carr	Metal
G9	Hex Nut (M4)	2	7.94	7.94	McMaster-Carr	Metal
G10	Spacer	4	0.93	3.72	McMaster-Carr	Metal
G11	Vibration-Damping Mount	3	14.00	42.00	McMaster-Carr	Composite

## 5 Build instructions

### 5.1 Mounting to grounding plate

The module was designed so that all components could be mounted on a plate prior to mounting the plate in the enclosure. This allowed for free access to all components to make all communications and power distribution connections while maintaining the small form-factor of the module. The grounding plate (W11) was designed to allow all components to fit inside the case (G1) in a layout that facilitated all power and communication connections while minimizing the gas flow path length to keep response time of the sensors high. There were three board-based component types that had the same geometry: NDIR sensors (S1 and S2), serial servers (C1 and C2), and 1/4 size soldered breadboards (W6). To keep the footprint of the module relatively small, these components were stacked on top of each other and connected using hex standoffs.

**Fig. 5:**
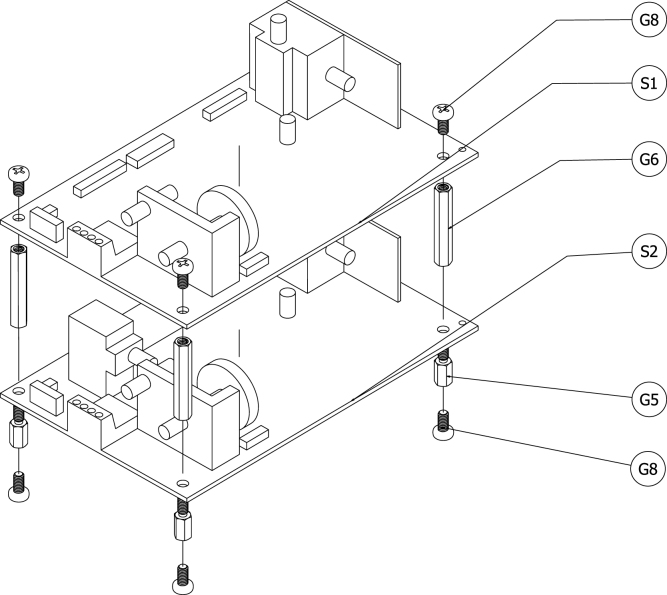
Schematic of NDIR sensor with mounting hardware.

Fig. 5 shows a schematic of the NDIR sensors and their associated mounting hardware. Four 10 mm long M4 hex standoffs (G5) were attached to the grounding plate (W11) by passing M4 machine screws (G8) through the holes in the grounding plate and into the female threaded side of the standoffs. The male threaded sides of the standoffs were inserted through the mounting holes at the corners of the CO NDIR sensor (S2) and four 35 mm long female–female M4 hex standoffs (G6) were screwed onto the male threads of the 10 mm long hex standoffs. The CO2 NDIR sensor (S1) was placed on top of the 35 mm standoffs and M4 machine screws (G8) were inserted through the sensor board mounting holes and into the 35 mm long standoffs to secure the sensor board to the standoffs.

**Fig. 6:**
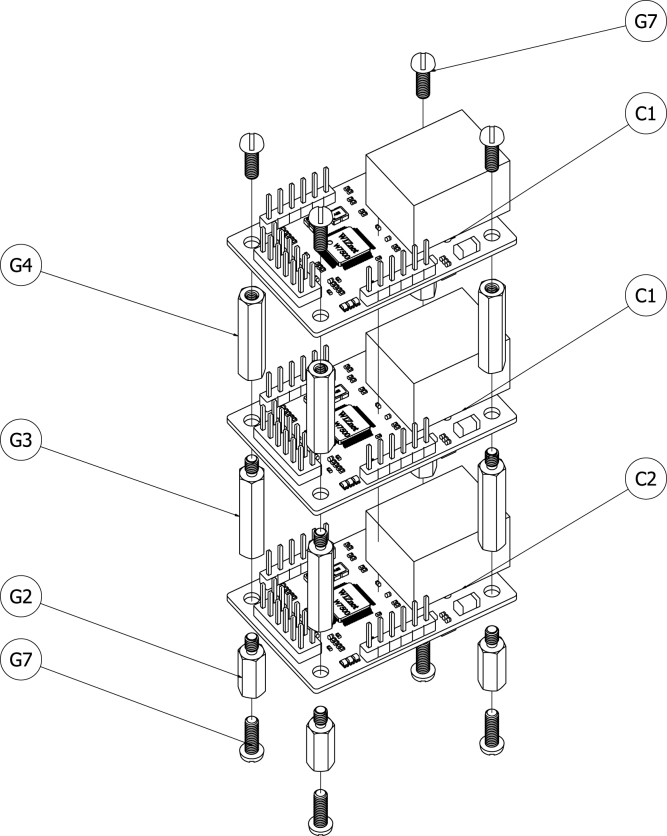
Schematic of serial servers with mounting hardware.

The same process was used to mount the serial servers to the grounding plate. Fig. 6 shows a schematic of the serial servers and their associated mounting hardware. Four 10 mm long M2.5 standoffs (G2) were attached to the grounding plate using M2.5 machine screws (G7). Female–male 20 mm long M2.5 standoffs (G3) separated the serial server closest to the grounding plate from the second serial server, and female–female 20 mm long M2.5 standoffs (G4) separated the second serial server from the highest serial server. Four M2.5 machine screws (G7) were used to secure the top serial server to the standoffs. The TTL serial server (C2) was installed closest to the grounding plate and the remaining two were RS-232 serial servers (C1). This arrangement was used to provide the easiest access between the NDIR sensors and the RS-232 servers.

**Fig. 7:**
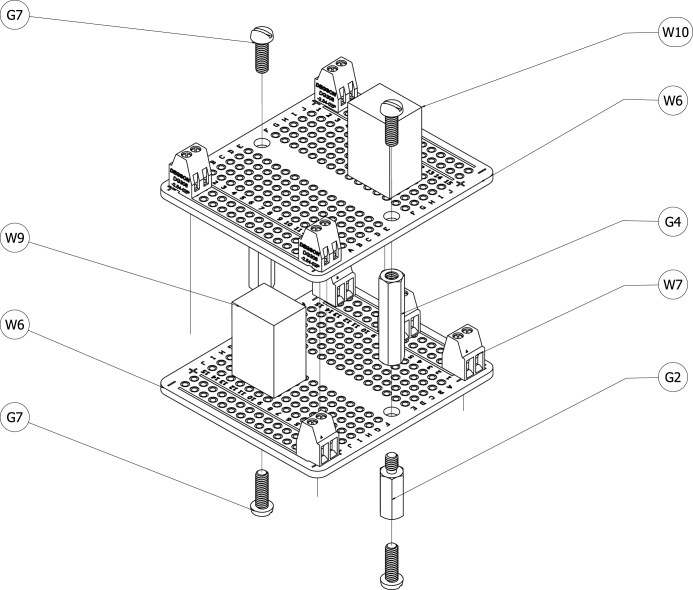
Schematic of soldered breadboards with mounting hardware.

The soldered breadboards (W6) were mounted in a similar way to the other board-based components. Fig. 7 shows a schematic of the serial servers and their associated mounting hardware. Two 10 mm long M2.5 standoffs (G2) were attached to the grounding plate using M2.5 machine screws (G7). Female–female 20 mm long M2.5 standoffs (G4) separated the lower breadboard from the higher breadboard. Two M2.5 machine screws (G7) secured the top breadboard to the standoffs.

**Fig. 8:**
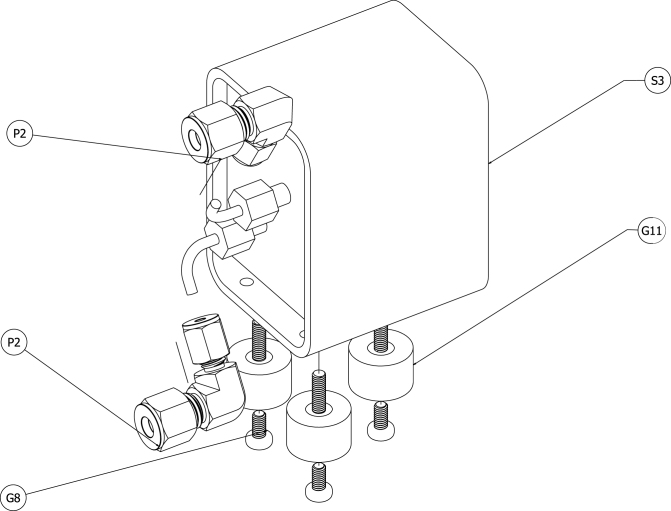
Schematic of O2 sensor, plumbing fittings, and mounting hardware.

Because of its measurement technology, the paramagnetic O2 sensor (S3) is highly sensitive to vibrations. This was one of the main reasons a pump was not directly incorporated into the sensor module. To limit the effect of vibrations on the measurement, vibration-damping mounts (G11) were used to attach the sensor to the grounding plate. The mounts were attached to the grounding plate with M4 machine screws (G8) from the bottom of the plate. Fig. 8 shows a schematic of the O2 sensor, the reducing elbows, and the mounting hardware.

**Fig. 9:**
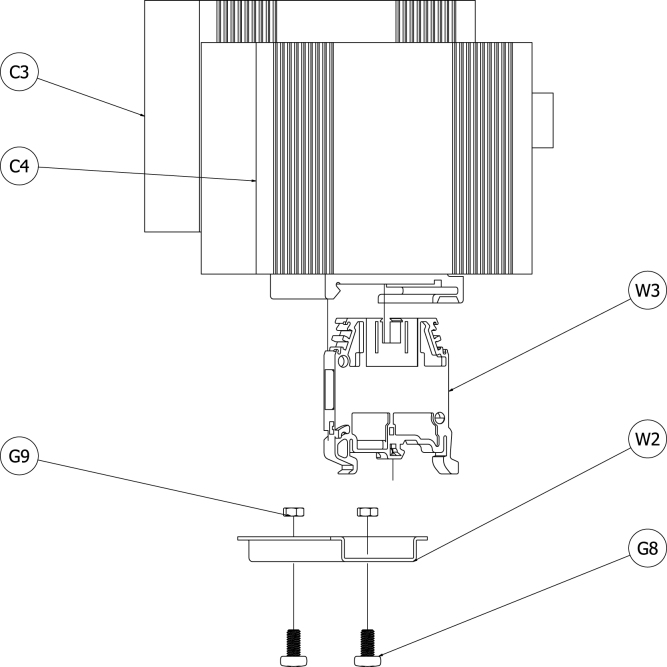
Schematic of PoE splitter, ethernet switch, and mounting hardware.

The PoE splitter (C3) and the ethernet switch (C4) are industrial equipment models and were shipped with DIN rail mounting hardware. A DIN rail (W2) was attached to the grounding plate with two M4 machine screws (G8) and secured with M4 hex nuts (G9). The PoE splitter and ethernet switch were mounted to the DIN rail such that the industrial terminal block for each was adjacent to the other. A grounding terminal block (W3) was also mounted on the DIN rail closest the edge of the grounding plate. Fig. 9 shows a schematic of the PoE splitter, ethernet switch, and mounting hardware.

### 5.2 Power distribution

**Fig. 10:**
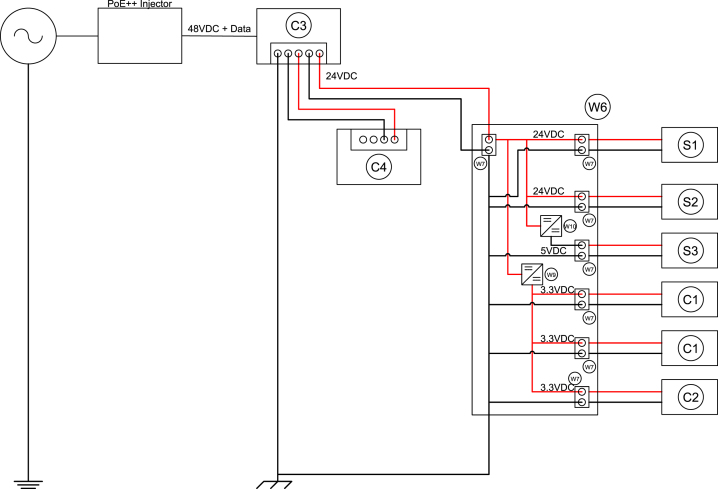
Schematic of power transmission connections and hardware.

Due to the power requirements of the hardware in the sensor module shown in Table 3, power transformation and distribution within the module is necessary. To minimize the space required to distribute power through the module, circuits were fabricated on soldered breadboards (W6). These soldered breadboards incorporate a terminal block (W7) used to energize the circuit with 24VDC. The circuits transform 24VDC to 5VDC and 3.3VDC using two step down regulators (W9 and W10) wired in parallel. Pre-cut jumper wires of various lengths (W8) were used to make soldered connections on the breadboards. A single terminal block was designated for each component that required external power. The 3.3VDC circuit had three output terminals, the 5VDC circuit had one output terminal, and the 24VDC circuit had two output terminals. The number of output terminals necessitated the use of two 1/4 size breadboards. An electrical schematic is provided in Fig. 10. A simple, custom printed circuit board could be designed and incorporated to reduce fabrication requirements and the footprint of the power distribution system, but was not undertaken for this design to limit the cost of the module.

The 3.3VDC output terminal blocks power the RS-232 serial servers (C1) and the TTL serial server (C2) [5]. The connection from the circuit terminal blocks to the serial servers is made using 6-inch female-to-female jumpers (W5). The 5VDC output terminal block powers the O2 sensor (S3) and the connection is made using the 12-inch female-to-female jumpers (W4). The two 24VDC output terminal blocks power the CO2 NDIR sensor (S1) and the CO NDIR sensor (S2) using 16 AWG stranded wire (W1) to connect the screw terminals of each board. All jumper connections to the board terminal blocks were made by stripping the end of the jumper and inserting the stripped wire into the screw terminal.

**Table 3:** Component electrical requirements.

Component	Supply voltage [VDC]	Power draw [W]
PoE splitter	24	3
Ethernet switch	24	6
Serial server	3.3	0.26
NDIR sensor	7–30	4
Paramagnetic sensor	5	0.5

The input to the distribution circuit is 24VDC and is connected to the industrial terminal connection on the PoE splitter (C3). 16 AWG stranded wire (W1) is used to connect the PoE splitter industrial terminal block to the industrial terminal block of the ethernet switch (C4). 16 AWG stranded wire is also used to connect the ground on the PoE splitter industrial terminal block to the grounding terminal block (W3) mounted on the DIN rail (W2). By grounding the DIN rail attached to the grounding plate, all components are electrically grounded and protected from electrical discharges or instabilities.

### 5.3 Communications

The NDIR sensors are capable of serial communication through the RS-232 communication protocol. A cable with the specific device pinout must be fabricated or purchased to connect the RS-232 port on the NDIR board to the serial server (C1) [6]. A 10-way Micro match free plug attached to a 10 way ribbon cable (C7) was used to make this connection. Pins 3, 5, and 9 were soldered to female jumpers that had been stripped on one end. Each soldered connection was sealed with heat shrink (C8) and the entire end of union between the ribbon cable and the jumpers was sealed with heat shrink (C8). The jumper attached to pin 3 (Tx) was connected to the RXD pin on the serial servers, pin 5 (Rx) was connected to the TXD pin on the serial servers, and pin 9 was connected to the GND pin on the servers.

The O2 sensor is capable of serial communication through a generic TTL communication protocol [7]. Female-to-female jumpers (W4) were used to connect pin P2 on the sensor (Tx) to the RXD pin on the serial server (C2) and pin P3 on the sensor (Rx) was connected to the TXD pin on the serial server. Accurate communication required that the O2 sensor and the serial server shared a common ground. Pin P4 (GND) on the O2 sensor was connected to ground on the soldered breadboard (W6), which was also connected to the GND pin of the server.

**Fig. 11:**
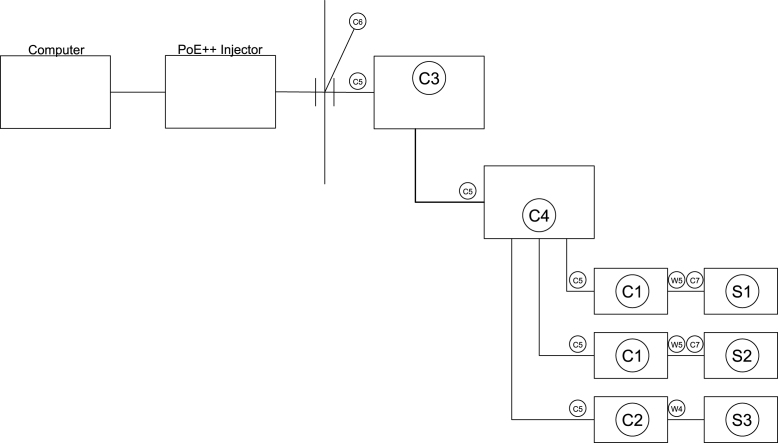
Schematic of communications connections and hardware.

Each of the serial servers was connected to the ethernet switch (C4) with a low profile CAT 6a ethernet cable with swiveling heads (C5). The upload port of the ethernet switch was connected to the data only port of the PoE splitter (C3). The PoE port of the PoE splitter was connected to the ethernet bulkhead fitting (C6) that was installed in the wall of the case (G1). A schematic of the communication connections is provided in Fig. 11.

To provide flexibility for interfacing between the computer and the sensor module, each serial server was established as a virtual communication (COM) port on the computer. This enabled the data to be retrieved via direct serial communication or by accessing the IP address of each serial server. Two applications were necessary to set up virtual COM ports: WIZnet Configuration Tool [8] and WIZVSP [9].

The Configuration Tool allowed all serial settings and the IP address of the server to be modified. A guide for configuring the serial servers is available from the manufacturer [10]. Prior to setting up the serial servers, the networking IP address of the computer used for interfacing with the sensor modules and for data acquisition was manually assigned with an IPv4 gateway of 192.168.11.1, an IPv4 address of 192.168.11.3, a DNS server of 8.8.8.8, and a subnet mask of 255.255.255.0. This assignment ensured that all devices with IP addresses in the range 192.168.11.X would be on the same network as the computer. In the configuration tool, each serial server was identified according to its MAC address. Each server was defined as a transmission communication protocol (TCP) server with a static network configuration that had a local IP address that was unique to each server and all other IP settings identical to those defined for the computer. The configuration tool was also used to specify that the server would listen on local port 5000. To simplify the process of mapping IP addressed to COM ports, the fourth octet of the IP address was defined as the COM port, e.g. 192.168.11.11 corresponded to COM 11. The serial settings were defined according to the sensor attached to the serial server. These settings for each sensor are shown in Table 4.

WIZVSP enabled the virtual COM port to be established on the computer. In the WIZVSP tool, a new client was created for each server and assigned the appropriate COM port number. Strict baud rate emulation was enabled and the remote IP that corresponded to the COM port was defined, e.g. 192.168.11.11:5000 for COM 11. The use of raw data transmission algorithms was selected in the communication preferences tab of the client definition. All other settings remained unchanged from the default. This set of tools and these configuration steps have only been tested on a Windows 11 operating system.

**Table 4:** Component communication settings.

Component	Baud rate [Hz]	Data bits	Stop bits	Parity	Flow control
NDIR sensor	57 600	8	1	None	None
Paramagnetic sensor	19 200	8	1	None	None

### 5.4 Plumbing

**Fig. 12:**
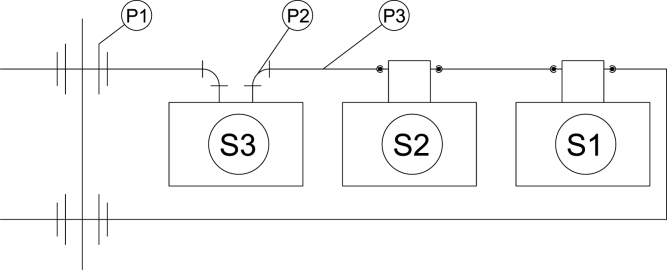
Schematic of plumbing connections and hardware.

A schematic of plumbing connections is provided in Fig. 12. Reducing elbow compression fittings (P2) were connected to the 1/8-inch inlet and outlet tubes on the O2 sensor (S3). 1/4-inch OD tubing (P3) was used to connect the elbow on the tube that was higher on the O2 sensor to the upper bulkhead fitting (P1) on the wall of the case. The elbow on the outlet tube of the O2 sensor was connected to the inlet barb fitting of the CO NDIR sensor (S2) with 1/4 OD tubing (P3). The outlet barb fitting from the NDIR sensor was connected to the inlet barb fitting of the CO2 NDIR sensor (S1) with 1/4 OD tubing (P3). The outlet barb fitting was connected to the lower bulkhead fitting (P1) on the wall of the case.

### 5.5 Assembly

**Fig. 13:**
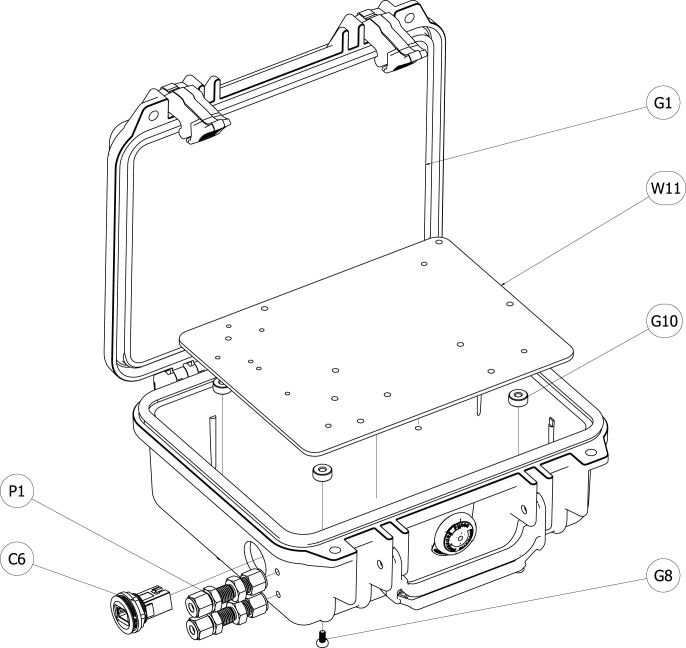
Schematic of case and grounding plate.

The case was prepared for assembly by drilling approximately 5 mm diameter holes through the bottom of the case in the centers of the annular feet of the case. To ensure the holes in the case lined up with the tapped holes in the grounding plate, the grounding plate was lain on the bottom of the case and a smaller diameter drill bit was used to drill pilot holes. The 5 mm bit was used to finish the holes after alignment was verified. 5 mm holes were also drilled in the wall of the case to make appropriate openings for the bulkhead plumbing fittings. A 27 mm hole saw was used to bore a hole through the wall of the case to accommodate the ethernet bulkhead fitting. Fig. 13 shows a schematic of the case and the plate mounting method.

All components were mounted to the grounding plate using hardware from beneath the plate in accordance with the procedures detailed in Section 5.1 and all electrical and communications connections were made in accordance with Sections 5.2, 5.3. Four spacers (G10) were placed collinear with the holes in the bottom of the case and the grounding plate was placed in the case on top of the spacers. Four M4 machine screws (G8) were used to secure the grounding plate to the case. The compression bulkhead fittings and the ethernet bulkhead fitting were installed in the wall of the case and the associated connections to bulkhead fittings detailed in Sections 5.4, 5.3 were made after the grounding plate was secured.

The ethernet port on the outside of the case was connected to a PoE++ injector that was compatible with the PoE splitter used in the sensor module. The PoE injector was connected directly to the computer when a single sensor module was utilized. When multiple sensor modules were used simultaneously, the data port from the PoE injector was connected to an ethernet switch. A multitude of solutions exist for powering the sensor modules and networking the communication cables to the modules, so a detailed description is only provided here for the single module case.

The O2 sensor incorporated into the sensor module has an upper flow rate limit of 200 mL/min. The gas does not need to be completely dry, but the humidity in the flow must be low enough such that it is non-condensing. A pump or pressurized system is necessary to drive flow through the sensor module and the module was intentionally designed without a pump to limit vibrations that would interfere with the measurements. A separate module that pumps, filters, and dries the sample gases was connected to the sensor module such that the pressure in the plumbing through the sensor module was slightly above atmospheric.

## 6 Operation instructions

The ethernet port in the wall of the case is connected to a PoE++ injector which is connected directly to a computer via an ethernet port. The inlet connection to the sensor module is connected to the sampling pump which is allowed to sample the gas mixture of interest. The sensor module outlet is connected to tubing that is directed to ventilation to ensure the user is not exposed to any products of combustion or calibration gas. When experiments are conducted in open air, no additional ventilation is necessary. Guidance from the sensor manufacturers indicate the sensors should be powered to allow them to warm up for approximately 30 min prior to data collection to ensure accurate and stable measurements.

The data acquisition and visualization script requires dependencies including python 3.12.0+, dash 3.2+, pyserial 3.5+, and plotly 5.24+. The script may be modified to change the COM ports that correspond to each sensor. Because the NDIR sensors return a fraction (0 to 1) of their nominal range (10% vol. concentration of target gas) as output, the script also allows the user to modify the range if a sensor of a different range is used.

**Fig. 14:**
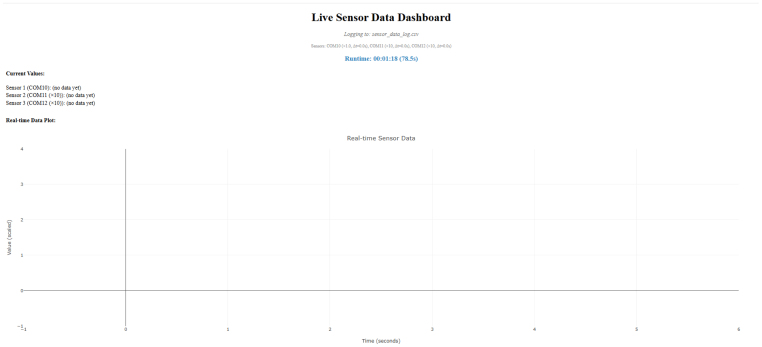
Screenshot of data visualization dashboard.

When the sensors are sufficiently warmed up, the python script is run from the command prompt, PowerShell, or terminal. The script launches a ‘dash’ server in the system default web browser (if the system is capable of launching a web browser) that streams the data from the sensor module at a rate of 2 Hz. The script also logs the data in a comma-separated values (CSV) file that is saved in the same directory as the script. A screenshot of the dashboard page is included as Fig. 14.

### 6.1 Environmental limitations

All components of the sensor module have limitations related to the sample gas properties and characteristics. The paramagnetic sensor has a maximum operating temperature and can tolerate a maximum sample gas temperature of 65 °C, a maximum humidity of 95%RH (non-condensing), a maximum particulate size within the gas of 3μm, and a maximum gas gauge pressure of 0.33 bar. The NDIR sensors have a maximum operating temperature of 45 °C, a maximum humidity of 99%RH (non-condensing), and an absolute gas pressure range of 0.800 to 1.150 bar. The serial servers have a maximum operating temperature of 70 °C and the ethernet switch has a maximum operating temperature of 75 °C. With these limitations in mind, it is important that moisture in the sample gas is non-condensing, the sample gas is filtered at least to 3μm, and the gas temperature does not exceed 45 °C or a gauge pressure of approximately 0.15 bar.

### 6.2 Safety considerations

There are some inherently hazardous aspects of measuring gas concentrations to characterize conditions in fire and combustion experiments. Byproducts of combustion, most prominently including carbon monoxide and soot, may be immediately dangerous if inhaled by a researcher conducting the experiment. Additionally, combustion is characterized by the rapid release of thermal energy that has the potential to injure the experimentalist or spread fire to nearby ignitable materials. When working with or in the vicinity of combustion and fire, it is prudent to be prepared with appropriate countermeasures (e.g. water hoses, fire extinguishers, etc.) and personal protective equipment (PPE) as deemed necessary for the experiment.

The sensor modules presented here target measurement of volumetric concentrations of oxygen, carbon monoxide, and carbon dioxide. Environments with reduced oxygen concentration and increased carbon monoxide and carbon dioxide concentrations may be hazardous. If experiments are not conducted in an open air environment, the exhaust from the sensor modules should be directed to exhaust ventilation and the experimentalist may consider including low-cost ambient air quality monitoring with alarms to indicate dangerously low oxygen concentrations or increased carbon monoxide conditions.

The sensor modules presented in this work introduce some additional safety concerns because they require compressed gas bottles for field calibration and incorporate electrical infrastructure to power the various components within the module. The compressed gas bottles and pressure regulators or valves should be inspected before use and bottles should be appropriately secured during transportation and use in experimentation. Safety glasses should be worn in the vicinity of compressed gases. If experiments are conducted in laboratory conditions, ventilation of fugitive emissions should be considered during experimental design.

Prior to supplying power to the sensor module, all electrical connections should be inspected to ensure all components are properly connected to the grounding plate (chassis ground) and that the grounding plate is properly connected to the earth ground at the PoE splitter. Any inspection of electrical components must be conducted when the sensor module is not energized and any charge on electrical components has been discharged. The lid of the sensor module should be closed whenever the module is energized. Because water is a product of combustion, it is possible that the gas line at the inlet of the sensor module contains condensed moisture. To prevent electrical short circuiting that could damage components of the module or that could be hazardous to the experimentalist, guidance concerning discharge of all electrical components should be strictly followed with awareness of the possibility of moisture exposure. Additional safety considerations related to specific components are included in manufacturer documentation [7], [11].

## 7 Validation and characterization

### 7.1 Calibration

Calibration for the NDIR sensors was conducted with ultra-high purity N2 to zero the sensors, and a gas mixture with nominal concentrations of 5%vol. CO, 10%vol. CO2, and balance N2 to calibrate the expected span of the sensors. Calibration of the O2 sensor involved ultra-high purity N2 to zero the sensor and either synthetic air (20.95%vol. O2) or ultra-high purity O2 to calibrate the span of the sensor, depending on the application and the expected oxygen concentration in the sample gas stream. All the sensors incorporated into the module are sensitive to the temperature and pressure of the gas flow, so it is important to calibrate the sensors under the same conditions that will be experienced during sample measurement. The ambient temperature and pressure as well as the temperature and pressure of the gases supplied to the sensor module during calibration should be recorded in case pressure and/or temperature compensation is necessary in post-processing the measured data.

The NDIR sensors incorporate a thermocouple and pressure transducer on the board and the signal from the transducer is used to compensate the signal for variations in temperature and pressure that have a non-negligible effect on measurements of the volumetric concentration. The temperature correction is described directly by the ideal gas law and the pressure correction is described by the ideal gas law as well as line broadening effects that disproportionately affect small molecules. The pressure correction is described by Eq. (1) [11], where $P_{0}$ is the nominal ambient pressure (1013.25 mbar) and P is the pressure measured at the NDIR pressure transducer (mbar).

(1) $$True\;\text{\%}V/V=Indicated\;Value\left[\frac{P_{0}}{1.5P-0.5P_{0}}\right]$$

The paramagnetic sensors have a response that is proportional to the volumetric concentration of oxygen in a gas mixture. The oxygen concentration reading from the paramagnetic sensor may be directly corrected due to the effect of pressure on the gas density. If the pressure of the gas flow is input to the sensor during calibration and a pressure reading is supplied to the sensor during the measurement, an internal pressure correction may be applied. The manual describes the steps required to allow for this internal pressure correction and recommends that the same correction be applied to the measurements externally if internal correction is not performed [7]. The pressure correction for the paramagnetic sensor is described by Eq. (2) where $P_{cal}$ is the gas pressure at the paramagnetic sensor during calibration and P is the instantaneous pressure at the sensor.

(2) $$\text{\%}O_{2,comp}=\text{\%}O_{2}\left[\frac{P_{cal}}{P}\right]$$

A pressure regulator was connected to each calibration gas cylinder and the outlet of each regulator was attached to a three-way diverting valve. The outlet of the diverting valve is connected to a module with a pump and a mass flow controller in line to drive and control the flow to the sensor module. The external module that pumps the sample gases into the sensor module must be capable of filtering and drying the gases to the appropriate criteria (presented in Section 6.1). The module must also be capable of supplying the gas at a flow rate of approximately 200 mL/min and tracking the temperature and pressure of the sample gases. The external sampling module used in this work incorporated a 15 µm sintered metal filter (Swagelok SS-4TF-15) and a 0.5 µm sintered metal filter (Swagelok SS-4TF-05), followed by a membrane gas dryer (Perma Pure PD-50T). The dried gas flows through a fitting with a relative humidity sensor (Innovative Sensor Technology HYT939P), then through a mass flow controller (Alicat MC-500SCCM). A pump (KNF NMP09KPDC-B 6V) that drives the flow is placed after the mass flow controller to ensure the flow through the sampling module is maintained at the set 200 mL/min setpoint.

To simulate conditions expected during gas sampling, the pressure regulators on the calibration gas cylinders were set to the lowest possible pressure that allowed flow (slightly above atmospheric pressure). The pump and mass flow controller were energized and the mass flow controller was set to allow 200 mL/min flow.

Procedures for serial communication with the sensors are outlined in application documents [12] and replicated briefly here to describe the calibration procedure. A terminal program (PuTTY) was used to connect to the virtual COM ports that corresponded to the NDIR and O2 sensors. The serial command ‘C1‹CR›’, where ‘‹CR›’ denotes a carriage return, signals to the NDIR sensors to enter calibration mode. When in calibration mode, the diverting valve is adjusted to allow the N2 to flow to the sensor module and the serial command ‘z’ is sent to the sensor to start the zero routine, which requires approximately 2 min. After the sensor is successfully zeroed, the span gas is applied to the sensor, the sensor is allowed to reach a steady state reading and the serial command ‘s‹value›‹CR›’ signifies to the sensor that the span value should be set according to ‘‹value›’, which is the fraction of the full range of the sensor (10%vol. for both NDIR sensors in the sensor module as described in this work).

The calibration procedure for the O2 sensor involves connecting to the sensor through a terminal program and flowing N2 into the sensor. The application note indicates the sensor should be allowed to stabilize with each calibration gas for at least 30 s prior to sending the command for the calibration routine. After the sensor reaches steady state, the serial command ‘0‹CR›’ signals to the sensor that the first calibration point corresponds to 0%vol. After the O2 sensor is successfully zeroed, the span gas (air or pure oxygen) is flowed to the sensor and the serial command ‘‹value›‹CR›’ signals to the sensor the oxygen concentration of the second calibration gas, where ‹value›is the %vol. of the span gas.

It is recommended that all sensors in the module are fully calibrated under the gas flow conditions consistent with the experiment before each test series. It is also recommended that at the outset of each day of testing, gases with known concentrations should be flowed through the gas sensor module to ensure the calibration is still valid in a process known as ‘bump testing’.

### 7.2 Response time

The response time of the sensors to a step change in the gas concentration is an important consideration in analysis that ensures the user is evaluating data from a sample that was collected at a known time. Because each gas sensor is in line in the flow-through orientation used in the sensor module, it is inevitable that there is a delay caused by the time required for the gas to flow through the tubing at the inlet of the module and through each successive sensor. Additionally, each sensor has a delayed response to a step input due to the physics of the sensing mechanisms and associated electronics. The data must be corrected by acknowledging these time delays and shifting the data accordingly.

The process adopted to determine the response time for the sensor modules involved flowing the zero gas (UHP N2) into the module until all sensors had a steady reading of approximately zero. When zero was achieved on all sensors, a gas sample was introduced that had non-zero values of each of the gases measured in the module (ambient air in this exercise). The time required for each sensor to achieve 90% of the steady state value (T90) was considered the response time for the specific sensor.

**Fig. 15:**
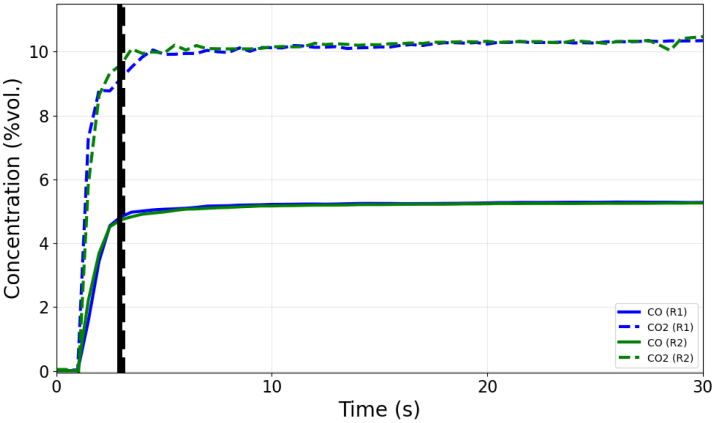
Gas concentration data collected in response time tests using calibration gas.

**Fig. 16:**
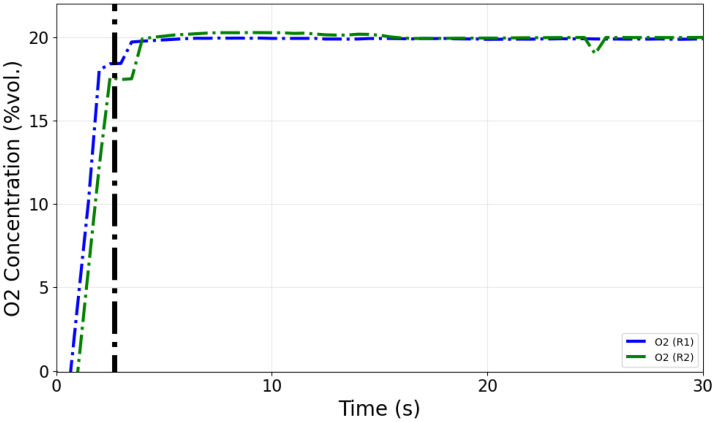
Gas concentration data collected in response time tests using environmental air.

A diverting valve was installed at the inlet of the sensor module to ensure the transition from the zero gas to the sample gas did not incorporate additional flow delay through connected tubing. Both the zero gas and the sample gas flowed through the sensor module with a flow rate of 200 mL/min. Fig. 15 displays data collected from duplicate cycles of a response time test using calibration gas (nominally 10%vol. CO2/5%vol. CO/balance N2). Fig. 16 displays data collected from duplicate cycles of a response time test which sampled air from the environment (nominally 20.95%vol. O2). The measured response time of the sensors is tabulated in Table 5 and indicated in Fig. 15, Fig. 16 with vertical lines. The observed response time values make sense intuitively based on the flow path from the O2 sensor to the CO and CO2 sensors.

**Table 5:** Observed sensor response times.

Gas	Response time (T90) [s]
O2	2.7
CO	2.9
CO2	3.1

### 7.3 Accuracy and repeatability

The response time, accuracy, and repeatability of the gas concentration measurements are dependent on the temperature, pressure, and flow rate of the gas sample being supplied to the sensor module. Ideally these conditions are identical between calibration and sampling, but in reality it is difficult or impossible to ensure consistent conditions in an experimental environment. The data from the experiments conducted to determine the response time of the gas sensors may also be analyzed to determine the accuracy and repeatability of the sensors under the specific conditions generated for those experiments.

The diverting valve was directed such that the sample flowed to the sensors and each gas was allowed to flow to the sensor modules for 180 s. The repeatability was defined as the mean instantaneous standard deviation of the signal from each sensor over the final 60 s of each cycle. The final 60 s were considered to ensure the flow and signals were steady. Table 6 displays the repeatability standard deviation of each sensor in the module. Each sensor shows high repeatability under the experimental conditions with less than 0.2% deviation between replicate cycles.

One experiment involved a standard calibration gas mixture, so its composition is documented and the other experiment involved sampling room air, which may have some variability but the oxygen concentration in atmospheric air is generally known. Testing with these well-characterized gas mixtures allows us to evaluate the accuracy of the sensors. Table 6 displays the mean error in each sensor measurement at steady state. Each sensor shows high accuracy under the conditions of the experiment with less than 5% deviation from concentrations of the known gas mixtures at steady state. The error in the concentration measurements may be attributed to changes in pressure when switching the flow from the zero gas to the sampled gas. The high repeatability of the measurements indicates that compensation for changes in the thermodynamic state of the gas samples and sampling conditions may be possible to give the user high confidence in the gas concentration measurements.

**Table 6:** Sensor signals at steady state.

Gas	Nominal	Repeatability	Steady error
	concentration	Std. Dev.	[%vol.]
	[%vol.]	[%vol.]	
O2	20.95	0.03	−1.09
CO	5.16	0.01	0.15
CO2	10.01	0.02	0.41

## CRediT authorship contribution statement

**Mark B. McKinnon:** Writing – original draft, Project administration, Investigation, Conceptualization. **Ian Brady:** Writing – review & editing, Validation, Investigation.

## Declaration of competing interest

The authors declare that they have no known competing financial interests or personal relationships that could have appeared to influence the work reported in this paper.
